# Heteroatom tri-doped porous carbon derived from waste biomass as Pt-free counter electrode in dye-sensitized solar cells†

**DOI:** 10.1039/c8ra02575d

**Published:** 2018-05-21

**Authors:** Pin Ma, Wenli Lu, Xiaoying Yan, Weidan Li, Li Li, Yanyan Fang, Xiong Yin, Zhengang Liu, Yuan Lin

**Affiliations:** State Key Laboratory of Chemical Resource Engineering, School of Science, Beijing University of Chemical Technology Beijing 100029 P. R. China yinxiong@mail.buct.edu.cn; Beijing National Laboratory for Molecular Sciences, Key Laboratory of Photochemistry, CAS Research/Education Center for Excellence in Molecular Sciences, Institute of Chemistry, Chinese Academy of Sciences Beijing 100190 P. R. China linyuan@iccas.ac.cn; CAS Key Laboratory of Standardization and Measurement for Nanotechnology, National Center for Nanoscience and Technology Beijing 100190 P. R. China; Laboratory of Solid Waste Treatment and Recycling, Research Center for Eco-Environmental Sciences, Chinese Academy of Sciences Beijing 100085 P. R. China zgliu@rcees.ac.cn; University of Chinese Academy of Sciences Beijing 100049 P. R. China

## Abstract

Strategies for environmentally friendly reutilization of waste biomass are highly desirable nowadays. Meanwhile, seeking Pt-free electrocatalysts for triiodide reduction with both high catalytic activity and low cost is critical for the development of dye-sensitized solar cells (DSCs). In the study, heteroatom tri-doped porous carbons (TPCs) were prepared *via* carbonization of a typical food waste (fish waste) and explored as a counter electrode (CE) in a DSC. The as-prepared carbon materials possessed a porous structure with a large BET surface area of 2933 m^2^ g^−1^, while being simultaneously naturally doped by three heteroatoms (N, P and S). More importantly, the resultant N, P, S-tri-doped porous carbon exhibited outstanding electrochemical activity towards triiodide reduction with good stability. Moreover, the DSC with the optimized TPC electrode showed a power conversion efficiency of 7.83%, which is comparable to the device with a costly Pt-based CE (8.34%), measured under one sun illumination (AM 1.5G). This work demonstrates that carbonization of fish waste offers a cost-effective approach to prepare multifunctional carbon materials for advanced energy applications.

## Abstract 2

Tri-doped porous carbon derived from waste-biomass was developed for a highly efficient counter electrode in dye-sensitized solar cell with an efficiency of 7.83%.

## Graphical abstract

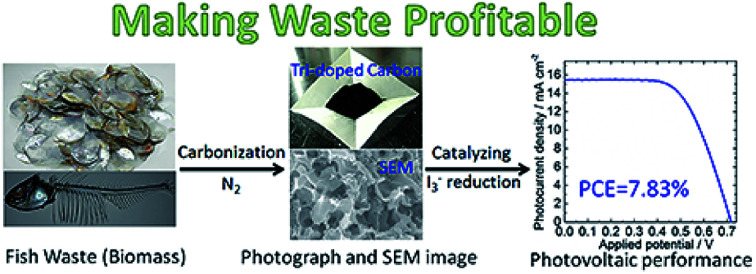

## 1 Introduction

Considering the growing demand of energy and worsening of environmental pollution, there is a must to develop sustainable energy systems.^1–3^ Dye-sensitized solar cells (DSCs) have attracted widespread attention in recent years due to their low cost, easy fabrication and relatively high power conversion efficiency.^1–9^ A typical DSC contains a dye-sensitized photoanode, liquid-state electrolyte containing the I_3_^−^/I^−^ redox couple and a counter electrode (CE).^4,6^ The redox reaction at the electrolyte/CE interfaces can influence the overall efficiency of the device. Consequently, the ideal catalyst for the CE should possess high conductivity, good electrocatalytic activity of the redox couple, excellent stability in the corrosive electrolyte and low production costs.^4,6^ In most cases, platinum metal is commonly utilized as a CE in DSCs due to its high conductivity and outstanding catalytic activity for triiodide reduction. However, its high-cost, scarcity and poor stability in corrosive I_3_^−^-based electrolyte limit its application in large-scale manufacture.^10–16^ Thus, alternatives to the Pt CE have been extensively explored, such as transition metal compounds,^14,17,18^ conducting polymers^19,20^ and carbon-based materials.^21–28^ Among these materials, the carbon-based materials, including mesoporous carbon,^22,27^ hollow carbon nanospheres,^23^ graphene^24–26^ and activated carbon,^28^ are the most popular electrode materials owing to their intrinsic chemical inertness against corrosive electrolyte as well as high surface areas. Further investigations reveal that the edges and defect sites within the graphitic carbon framework provide active sites for efficient triiodide reduction.^21–23,25,26^ Moreover, the implantation of heteroatoms, such as nitrogen, sulfur and phosphorus, into the graphitic carbon lattice could induce charge redistribution, create more active sites, and thus boost the reduction of the redox couple.^21–23,26,29^ For example, the lone pair electrons in both pyridinic and quaternary nitrogen states account for promoting the catalytic activity of disulfide/thiolate redox shuttle and cobalt trisbipyridine redox mediator.^29–31^ With phosphorus-doping treatment on the reduced graphene oxide (rGO) CE, the power conversion efficiency of the device increased from 4.18 to 6.04%.^21^ The enhanced catalytic performance is ascribed to the existence of P–C bonds distributed in the carbon matrix.^21^ Meanwhile, dual-doped carbonaceous materials have also been widely investigated as counter electrode materials in DSCs.^30^ Compared with the N-doped (3.85%) or S-doped (4.23%) rGO CE-based device, the device containing N, S dual-doped rGO CE showed an increased power conversion efficiency (4.73%).^30^ The increased performance is attributed to the presence of a highly localized state of carbon within the carbon frameworks. This enormous progress indicates that carbon-based materials are promising alternative materials for Pt CE in DSCs.

Generally, the above-mentioned porous carbon materials were prepared using soft or hard template approaches with chemicals containing nitrogen and sulfur as the heteroatom sources. Additionally, the synthesis strategy involves rigorous preparation conditions and high production cost. Therefore, simply and cost-effective approaches are desirable for synthesis of porous carbon catalysts for high-efficient counter electrode. Biomass is a low-cost raw material for preparing carbon materials because of its rich organic functional groups, abundant supply and environmental friendliness.^32–34^ Consequently, much effort has been devoted to the preparation of functional carbon materials from renewable biomass for energy applications. For instance, pine cone flower and sea tangle were utilized to synthesize porous carbons,^11,35^ and soybean powder was used to prepare carbon quantum dots in photovoltaic applications.^33^ As reported, a large amount of fish waste is generated in the food industry daily. Fish waste is abundant in nitrogen, sulfur and phosphorous elements, besides having a high content of carbon.^36^ Thus, fish waste is an ideal precursor for synthesis of heteroatom-doped carbon materials. Nevertheless, to the best of our knowledge, the heteroatom doped porous carbon derived from biomass as a counter electrode in DSCs has never been investigated.

Herein, in the present study, we demonstrated the synthesis of tri-heteroatom-doped porous carbon (TPC) for DSC application *via* simple carbonization of fish waste. As-prepared TPC possessed a large surface area. Moreover, the TPC electrode presented both outstanding catalytic activity and high electrochemical stability on triiodide reduction. Besides, the synthesis process is simple and easy to control, and thus suitable for industrial application. These interesting results demonstrated that porous carbon derived from fish waste exhibited advantages for application as a high-performance counter electrode in DSCs.

## 2 Experimental

### 2.1 Chemicals

Potassium hydroxide, lithium perchlorate, hydrochloric acid, ethyl cellulose, terpineol and titanium isopropoxide in analytical grade were from Sinopharm Chemical Reagent Co. Ltd. Lithium iodide, iodine, dimethylpropyl imidazolium iodide, H_2_PtCl_6_, *tert*-butylpyridine and acetonitrile were purchased from Sigma-Aldrich Chemical Inc. These chemicals and solvents were in analytical grade and used as received.

### 2.2 Preparation of carbon counter electrodes

The porous carbon was prepared from fish waste according to reported processes.^36^ The fish scales were washed with de-ionized water, and then dried in an oven. The raw material was firstly pre-carbonized at 300 °C for one hour under nitrogen atmosphere. Subsequently, the pre-carbonized sample was activated with KOH in a quartz boat at 700 °C for 1.5 hours under nitrogen atmosphere. The as-obtained solid sample was washed with diluted HCl aqueous solution and de-ionized water, respectively. Finally, the product was dried at 80 °C for 24 h and kept in desiccator before use.

To fabricate carbon counter electrode, the as-obtained material was firstly made to paste.^10^ Briefly, a certain amount of carbon powder (0.05 g, 0.08 g and 0.1 g) was grinded with 1.0 mL of binder solution under atmosphere and stirred for 24 h to get a homogeneous dispersion. The binder solution was the mixture of 0.2 g of ethyl cellulose and 8.0 mL of terpineol as well as 0.5 mL titanium isopropoxide. Then, the carbon pastes were coated on the FTO-glass (sheet resistance: 15 Ω sq^−1^, Nippon Sheet Glass Co., Japan) using doctor-blading method and dried at 120 °C for 15 min. The electrodes were subsequently sintered under Ar atmosphere at 500 °C for 30 min. The thickness of the porous carbon layer on the resultant electrode was estimated using SEM. For comparison, the Pt/FTO CE electrode was prepared by thermal decomposition of H_2_PtCl_6_ (5 mM in isopropanol) on the FTO substrate using spin-coating method and sintered at 390 °C for 15 min.

### 2.3 Device assembly

The TiO_2_ film consisted of a transparent TiO_2_ layer (particle size of 20–30 nm, thickness of 7.2 μm, (Ti-Nanoxide HT/SP)) and a scattering layer (a mixture of 200–400 nm rutile TiO_2_ particles, thickness of 5.8 μm, Dyesol (WER4-O)).^19^ The DSC device had a sandwich-type configuration with a N3-sensitized TiO_2_ electrode, a counter electrode and liquid electrolyte. The electrolyte was 0.1 M lithium iodide, 0.05 M iodine, 0.6 M dimethylpropyl imidazolium iodide and 0.5 M *tert*-butylpyridine in acetonitrile. The N3 photoanodes were prepared by immersing TiO_2_ electrode into 0.5 mM N3 dye solution for 24 hours.

### 2.4 Characterization

The morphologies and EDX mapping of the as-prepared carbon samples were observed with a field emission scanning electron microscopy (SEM, Hitachi SU-8010) and transmission electron microscopy (TEM, Hitachi HT7700). X-ray photoelectron spectroscopy (XPS) measurement was carried out with an ESCA Lab 250xi spectrometer using Al Kα (1486.6 eV) irradiation as X-ray source. All the spectra were calibrated to the binding energy of the adventitious C 1s peak at 284.8 eV. Raman spectrum was recorded using a Renishaw inVia spectrometer. The porosity and BET surface area were obtained from nitrogen adsorption isotherms at 77 K using a Micromeritics 3Flex analyzer (USA). Prior to N_2_ sorption analysis, the sample was degassed at 300 °C for 12 hours.

Cyclic voltammogram (CV) measurement was performed in a three-electrode cell with liquid electrolyte containing 0.1 M LiClO_4_, 10 mM LiI, and 1 mM I_2_ in acetonitrile. The carbon electrode or Pt electrode was used as the working electrode, a saturated calomel electrode (SCE) served as the reference electrode, and platinum wire as the counter electrode. The photocurrent density–voltage (*J*–*V*) of device was performed using a digital source meter (Keithley 2611, USA). A 300 W Xe arc lamp (Oriel) equipped with optical filters was used as a light source for simulating the solar spectrum at AM 1.5 (100 mW cm^−2^). The intensity of light was calibrated with a Si solar cell. Electrochemical impedance spectroscopy (EIS) of dummy cells was measured using a Solartron SI 1287 electrochemical interface and a Solartron 1255B frequency response analyzer in the frequency range from 0.05 to 10^5^ Hz. EIS spectrum for DSCs with different electrodes were obtained using the same frequency response analyzer and potentiostat at amplitude of 10 mV and the open-circuit voltage under light irradiation of 100 mW cm^−2^ in the frequency range from 0.05 to 10^5^ Hz. The obtained data was fitted with a Z-View software.

## 3 Results and discussion

**Fig. 1:**
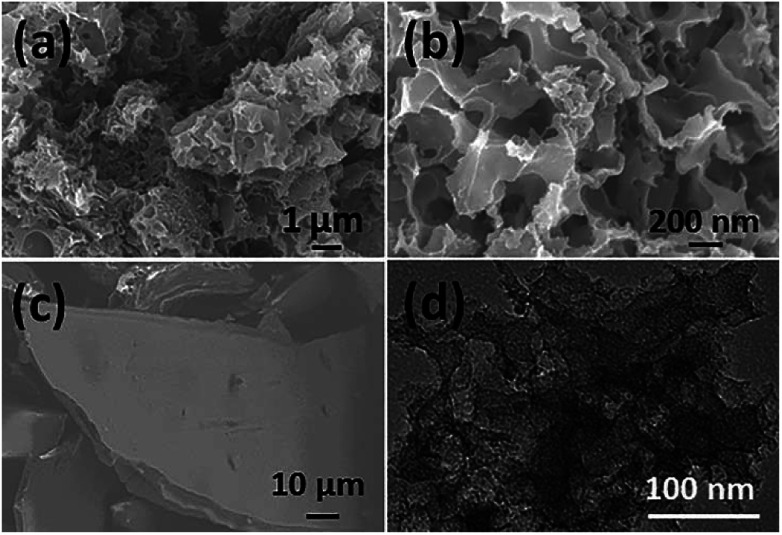
SEM images of as-obtained porous carbon sample prepared at 700 °C (a and b) and fish waste pre-carbonized at 300 °C (c); (d) typical TEM image of porous carbon sample.

The morphologies of the as-obtained sample (denoted as TPC) prepared at 700 °C and the pre-carbonized fish sample were characterized with scanning electron microscopy (SEM) and transmission electron microscope (TEM). The corresponding SEM and TEM images are shown in Fig. 1. It can be clearly found that numerous pores were present in the as-obtained carbon sample, with a honeycombed morphology (Fig. 1a and b). In addition, large amount of pores formed a 3D meshy structure. Whereas, no obvious pores were observed in the case of pre-carbonized fish sample (Fig. 1c). Therefore, the 3D porous structure was formed during the carbonization process at 700 °C. The formation of abundant pores was due to the activation by KOH and removal of hydroxyapatite. The hydroxyapatite also acted as the self-template during the pyrolysis process. Furthermore, its corresponding TEM image (Fig. 1d) also confirms that there were abundant pores formed in the sample.

**Fig. 2:**
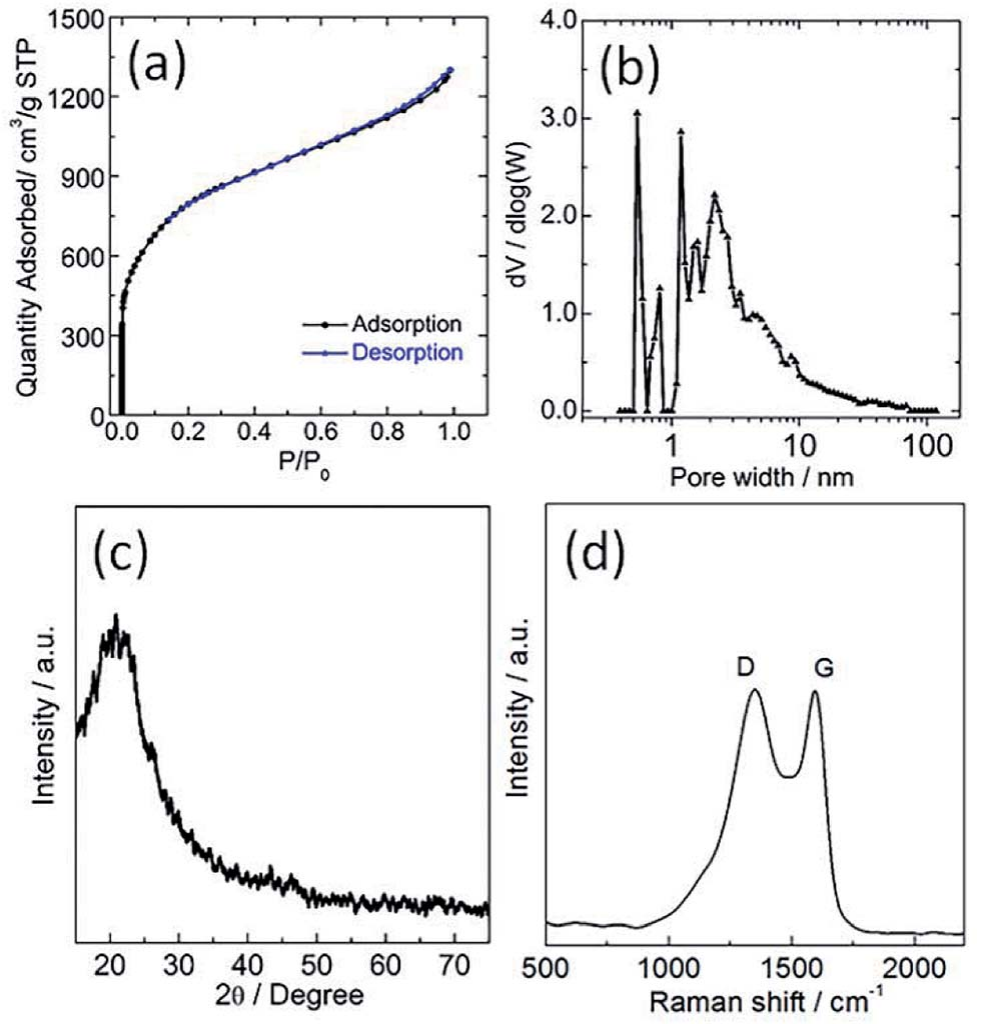
(a) Nitrogen adsorption/desorption isotherms, (b) its corresponding pore size distribution (c) XRD pattern and (d) Raman spectra of as-obtained porous carbon sample.

The sample was investigated using N_2_ physisorption measurements to provide insight into the micro- and meso-pore structures. The N_2_ adsorption/desorption isotherms and its corresponding pore size distribution curve of the sample are shown in Fig. 2a and b, respectively. The N_2_ adsorption/desorption isotherms of the sample showed a steep increase at a low relative pressure (*i.e.*, *P*/*P*_0_ < 0.01) as a result of filling of the micropores, and a hysteresis loop was observed at a high relative pressure range (*i.e.*, 0.6 < *P*/*P*_0_ < 1) ascribed to the capillary condensation of N_2_ inside the mesopores. Its corresponding specific BET surface area and total pore volume are 2933 m^2^ g^−1^ and 0.81 cm^3^ g^−1^, respectively. The pore size distribution of TPC sample calculated using DFT method from N_2_ adsorption isotherm is about 2.7 nm (Fig. 2b). These results also confirm that hierarchical porous structure and ultrahigh surface area were simultaneously obtained for the sample *via* carbonization process, which are in good agreement with SEM and TEM observation. The as-obtained porous structure will promote the mass transport, and increase the number of active sites for tri-iodide reduction reaction, and thus improve the catalytic performance.^10,27^ Shown in Fig. 2c and d are X-ray diffraction (XRD) patterns and Raman spectra of the as-prepared TPC sample, respectively. Only one diffraction peak can be observed at value of 23.1°, assigned to the (002) plane of graphene, indicating high purity of sample without other impurities. The Raman spectra of TPC had two characteristic peaks, located at 1350 and 1590 cm^−1^, which are ascribed to the characteristic D and G bands of graphitic carbon materials, respectively.^29^ Additionally, the value for intensity ratio of D and G band (*I*_D_/*I*_G_) is about 1.0, implying good graphitic degree of as-prepared sample.^29^ Results from XRD and Raman indicate that the fish waste was successfully converted into graphitized carbon without other impurities *via* a simple pyrolysis process.

**Fig. 3:**
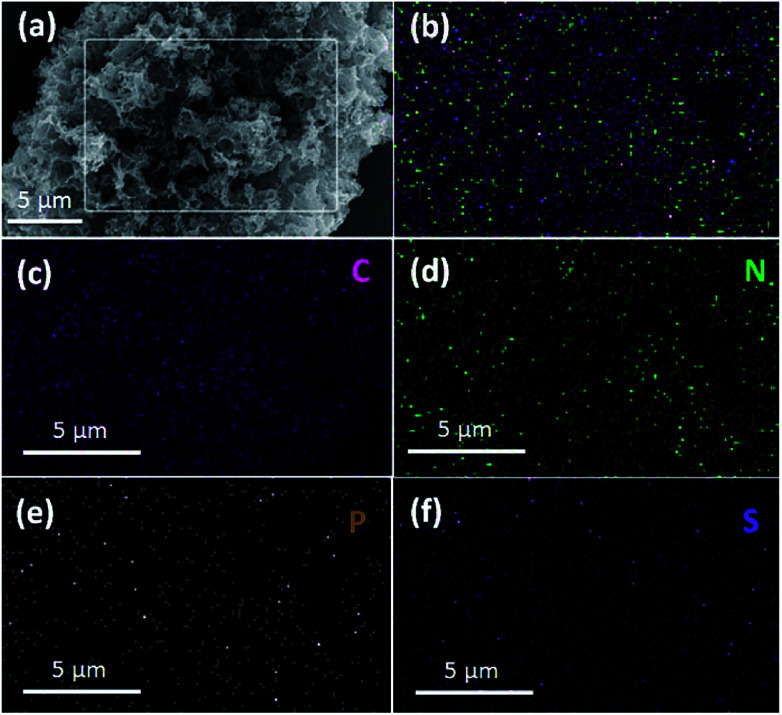
SEM image (a) and typical elemental mapping image (b) for as-obtained porous carbon sample with corresponding elemental mapping images of C (c), N (d), P (e) and S (f) elements.

**Fig. 4:**
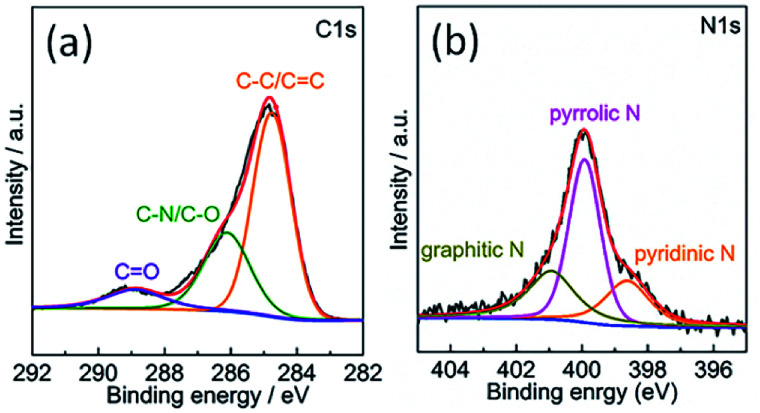
XPS core level spectra of (a) C 1s and (b) N 1s of the surface of as-obtained porous carbon sample.

The chemical composition of the TPC material was investigated with energy-dispersive spectroscopy (EDS). The typical EDS mapping images are shown in Fig. 3. Four elements, including C, N, P and S, were observed, with homogeneous distribution in the whole zone. Oxygen element was also present in the image, due to the exposure under the ambient atmosphere and derivation from fish scales (Fig. S1†). The TPC sample was also characterized with X-ray photoelectron spectroscopy (XPS) to understand its surface chemical states. The survey spectrum is given in Fig. S2.† As expected, the C, N, P and S elements were detected from the surface of sample, which is in good agreement of result from elemental mapping analysis. In addition, O was also detected, in accordance with results of EDS characterization (Fig. S1†). The core level spectrum of C 1s is shown in Fig. 4a. It can be disintegrated into three subpeaks located at 284.6, 286.1 and 289.2 eV, respectively. The strong peaks at 284.6 and 286.1 eV are associated with the graphite-like sp^2^ C and C 1s state in C–N/C–O, respectively.^29,37^ The low subpeak at 289.2 eV is attributed to formation of C=O bond. Meanwhile, the core level spectrum of N 1s can be deconvoluted into three subpeaks at 398.6, 399.9 and 400.9 eV, as shown in Fig. 4b. The subpeaks at 398.6, 399.9 and 400.9 eV are signals of pyridine-like, pyrrole-like and graphitic N, respectively.^37^ The presence of pyridine-like and graphitic N are thought to promote the catalytic activity.^26^ Furthermore, weak signals from P and S elements were also detected. Core level spectrum of P 2p and S 2p are shown in Fig. S2b and c,† respectively.^21,22^ The formation of C–S–C and P–C bonds will also promote the catalytic performance.^21,22^ Combining the results of XPS and those from XRD and EDS characterizations, it can be confidently concluded that the N, P and S elements were successfully doped into porous carbon matrix. The estimated atomic content of N, P and S is about 7.9, 1.2 and 1.2%, respectively. Doping carbon materials with ternary heteroatoms will benefit the catalytic activity on triiodide reduction process.^29–31^

**Fig. 5:**
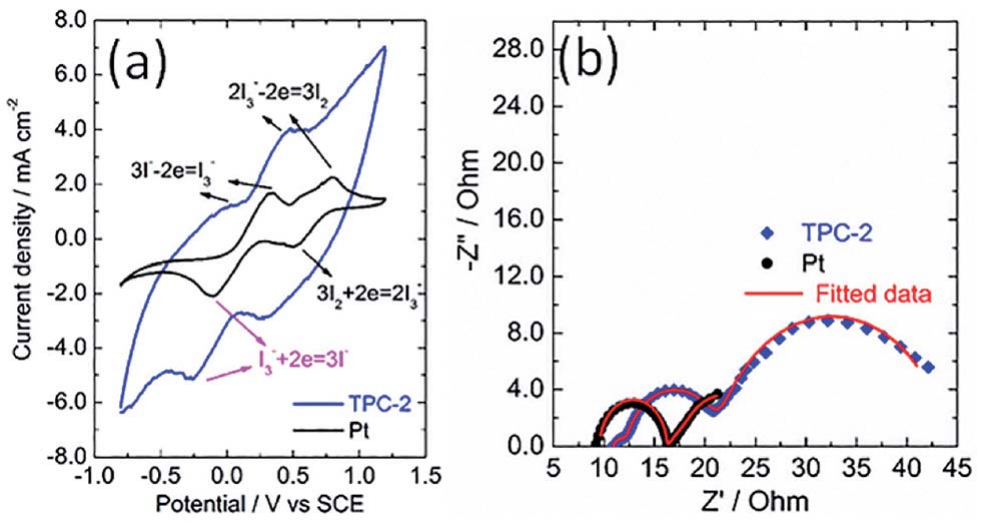
(a) Cyclic voltammograms for TPC and Pt electrode at a scan rate of 100 mV s^−1^ in acetonitrile solution containing 10 mM LiI, 1 mM I_2_, and 0.1 M LiClO_4_ with a platinum wire and a SCE electrode used as counter electrode and reference electrode, respectively; (b) Nyquist plots for electrochemical impedance spectra of the symmetrical cells with two identical TPC-2 and Pt electrodes.

The catalytic activity of the as-obtained TPC carbon towards triiodide reduction was evaluated with cyclic voltammetry (CV) analysis, in comparison with that of Pt electrode. Fig. 5a shows the CV curves of TPC and Pt electrodes in the acetonitrile solution containing LiClO_4_ as the supporting electrolyte, with LiI and I_2_ as the redox couple. Two pairs of oxidation/reduction peaks were observed for Pt electrode, whereas, one typical pair of I_3_^−^ ion oxidation/reduction peaks was present in the case of TPC carbon electrode within scanning range. During the operation of dye-sensitized solar cells, the produced I_3_^−^ ions must be efficiently reduced to I^−^ ions at the CE interface.^1–6^ Thus, the reduction peak of triiodide reduction is the research focus of CV analysis.^18,20^ The cathodic peak potential for TPC electrode is very close to that for Pt electrode. As expected, the cathodic peak current density of TPC electrode is much larger than that in Pt electrode. This is due to large surface area of TPC electrode, compared with Pt CE. These results imply that the as-prepared TPC electrode can effectively catalyze I_3_^−^/I^−^ redox couple, similar to the case of Pt electrode. The CV curves for two electrodes were recorded with 100 cycles to check the electrochemical stability. The changes in cathodic and anodic current density for two electrodes are summarized in Fig. S3.† In the case of Pt electrode, the final cathodic and anodic current density is 73% and 59% of initial values after 100 cycles scanning. Whereas, as for TPC electrode, the final values are about 90% of initial values. The results demonstrate that TPC electrode possessed good stability in corrosive I_3_^−^-based electrolyte, surpassing that of Pt electrode.

The electrochemical characteristics of CE was also investigated with electrochemical impedance spectra conducting on a symmetric sandwich device configuration with two identical electrodes.^24,25,39^ The Nyquist plots for TPC and Pt electrodes are displayed in Fig. 5b. Two arcs were present for Pt electrode, however, three semicircles were observed in the case of TPC electrode. The presence of three semicircles is due to the porous nature of electrode materials.^19,24,25^ The equivalent circuit for fitting experimental results is listed in Scheme S1.† The fitting data of the charge transfer resistance (*R*_ct_) for Pt and TPC electrode are 0.42 and 0.54 Ω cm^2^, respectively. Obviously, the two electrodes exhibited nearly identical *R*_ct_, which is largely below than 10 Ω cm^2^ needed for highly-efficient dye-sensitized solar cells.^19^ These results indicate that TPC could be used as efficient counter electrodes for dye-sensitized solar cells.

**Fig. 6:**
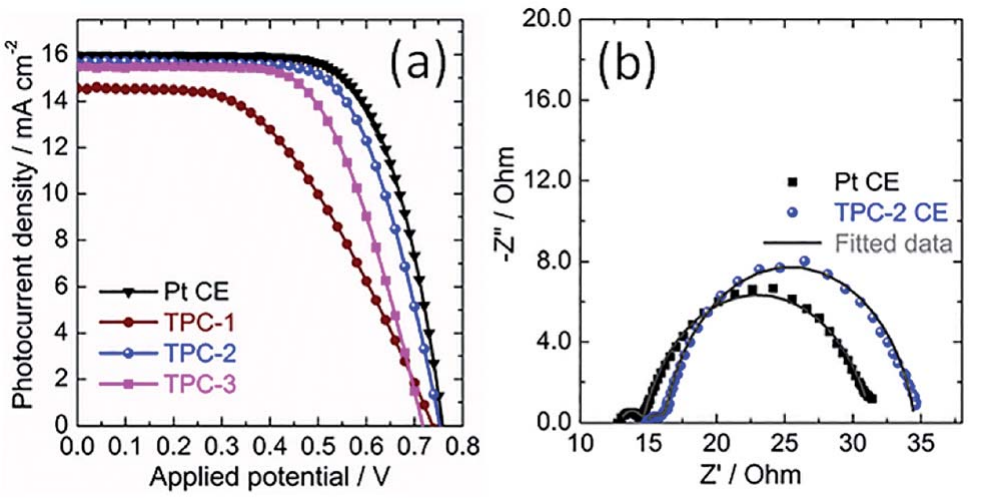
(a) Characteristic photocurrent density–voltage curves of the DSCs with TPC CEs of different thicknesses and Pt CE, measured under solar simulator illumination of 100 mW cm^−2^ (AM 1.5G); (b) electrochemical impedance spectra of DSCs using TPC-2 and Pt CEs measured under light irradiation, at open-circuit voltage; dots present experimental results, and solid lines show fitted results.

Consequently, the TPC electrodes of different thicknesses and N3-sensitized TiO_2_ photoanodes were used to assemble solar cells. For comparison, the DSC containing conventional Pt CE was also fabricated as reference. The corresponding photocurrent density-voltage curves of devices are shown in Fig. 6a, with photovoltaic parameters summarized in Table 1. Obviously, the power conversion efficiency (PCE) of DSCs is dependent on the thickness of TPC deposited on the electrode. Initially, the PCE of device enhanced with thickness increase from 8.8 to 11.6 μm. However, with further enhancing the thickness to 13.2 μm, the efficiency of device declined to 6.91%. The initial increase in photovoltaic performance with increasing thickness might be due to the increased active area for triiodide reduction, which can promote the charge transfer at the electrolyte/electrode interface. However, as thickness of carbon CE increases further, the resistance may increase and the diffusion of redox couple within the porous film would become more and more difficult, resulting in the decline in photovoltaic performance. As a result, among the three TPC-based solar cells, device with TPC-2 CE exhibited the highest PCE (7.83%), with an open-circuit photovoltage (*V*_oc_) of 0.750 V, a short-circuit photocurrent density (*J*_sc_) of 15.64 mA cm^−2^, and fill factor (FF) of 0.668. The as-optimized photovoltaic performance for TPC-based device cloud be comparable to that for conventional Pt-based device (PCE of 8.34%, *J*_sc_ of 15.98 mA cm^−2^, *V*_oc_ of 0.755 V and FF of 0.691).

**Table 1:** Photovoltaic parameters of DSCs with TPC and Pt CEs, measured under simulated solar illumination of 100 mW cm^−2^ (AM 1.5G)

Counter electrode	*J*_sc_/mA cm^−2^	*V*_oc_/V	FF	PCE/%
Pt	15.98	0.755	0.691	8.34
TPC-1	14.55	0.735	0.485	5.19
TPC-2	15.64	0.750	0.668	7.83
TPC-3	15.46	0.715	0.625	6.91

The photovoltaic properties of devices with different CEs were investigated using the electrochemical impedance spectra (EIS).^19,39–42^ EIS of DSCs with TPC-2 and Pt CEs was measured under the light illumination (100 mW cm^−2^, AM 1.5G). The corresponding Nyquist plots are presented in Fig. 6b. Two arcs were observed in the Nyquist plots for both TPC and Pt CE-based devices. According to previous reports, the high-frequency arc is due to the charge-transfer resistance at the interface of CE/electrolyte (*R*_CT1_), and the arc at middle frequency is attributed to the charge transfer resistance at the interface of N3-sensitized TiO_2_/electrolyte (*R*_CT2_). Meanwhile, the low frequency semicircle is ascribed to the diffusion resistance of redox couple within the electrolyte (*Z*_N_). In most cases, the *R*_CT2_ are commonly overlapped with *Z*_N_ due to the application of liquid state electrolyte in the study.^19,38^ The fitted curves with the equivalent circuit are also shown in the Fig. 6b. The fitted values of *R*_CT1_ for TPC-2 and Pt-based devices are 1.58 and 1.52 Ω, respectively. The almost equal values of *R*_CT1_ for both devices confirm that the as-prepared CE (TPC-2) could catalyze I_3_^−^/I^−^ redox couple as efficiently as Pt CE. The corresponding values of *R*_CT2_ are 18.21 Ω for TPC-2 CE and 16.81 Ω for Pt CE. The DSC with a TPC-2 CE showed a slightly larger *R*_CT2_ than that for Pt-based solar cell. The power conversion efficiency of device is dependent on the total resistance of the device.^19,38^ Therefore, *R*_CT1_ and *R*_CT2_ in the case of TPC-2 electrode can lead to a slightly low FF for TPC-2 based devices, compared to device with a Pt CE. The high performance for device containing TPC CE could be ascribed to high surface area and well-defined porosity for promoting electrolyte diffusion within electrode, as well as heteroatom doping-induced electrocatalytic activity on I_3_^−^ reduction.

## 4 Conclusions

Ternary heteroatom-doped porous carbons were successfully prepared *via* pyrolysis approach using fish waste as raw material in an inert atmosphere. The N, P and S elements contained in the fish waste were doped simultaneously into porous carbon matrix during the pyrolysis process. The resultant porous carbons possessed both large surface area and highly graphitized nanostructures, and thus presented perfect catalytic activity on triiodide reduction. The optimized DSC with a TPC-2 CE exhibited a power conversion efficiency of 7.83%, which is comparable to that for the device with a Pt CE (8.34%). The results indicate that porous carbon derived from fish waste could catalyze as efficiently as noble metal Pt on the triiodide reduction in DSC. The idea of “making waste profitable” reported here could be suitable for exploring low-cost non-noble metal catalysts in a wide variety of applications.

## Conflicts of interest

There are no conflicts to declare.

## Supplementary Material

RA-008-C8RA02575D-s001
