# Supplementary material for: Heteroatom tri-doped porous carbon derived from waste biomass as Pt-free counter electrode in dye-sensitized solar cells
Source: RSC Adv. 2018 May 21;8(33):18427–33. doi: 10.1039/c8ra02575d (PMC9080547; doi:10.1039/c8ra02575d)
Supplement: RA-008-C8RA02575D-s001 [file RA-008-C8RA02575D-s001.pdf]

Electronic Supplementary Material (ESI) for RSC Advances.
This journal is © The Royal Society of Chemistry 2018

Electronic supplementary information (ESI)

Heteroatom Tri-doped Porous Carbon Derived from Waste Biomass as Pt-free Counter Electrode in Dye-sensitized Solar Cell

Pin Ma [b,e,‡], Wenli Lu [a, ‡], Xiaoying Yan [c,‡], Weidan Li [a], Yanyan Fang [b], Xiong Yin [a,*], Zhengang Liu [d,*], Yuan Lin [b,*]

[a] State Key Laboratory of Chemical Resource Engineering, School of Science, Beijing University of Chemical Technology, Beijing 100029, P. R. China

[b] Beijing National laboratory for Molecular Sciences, Key Laboratory of Photochemistry, CAS Research/Education Center for Excellence in Molecular Sciences, Institute of Chemistry, Chinese Academy of Sciences, Beijing 100190, P. R. China

[c] CAS Key Laboratory of Standardization and Measurement for Nanotechnology, National Center for Nanoscience and Technology, Beijing 100190, P. R. China

[d] Laboratory of Solid Waste Treatment and Recycling, Research Center for Eco-Environmental Sciences, Chinese Academy of Sciences, Beijing 100085, P. R. China

[e] University of Chinese Academy of Sciences, Beijing 100049, P. R. China

---

‡ These authors contributed equally to this work.

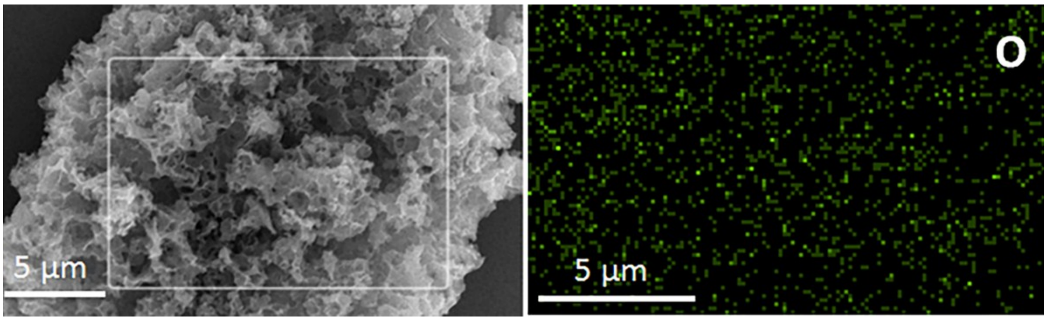

Figure S1. SEM image (a) and elemental mapping image (b) of O element for as-obtained porous carbon sample.

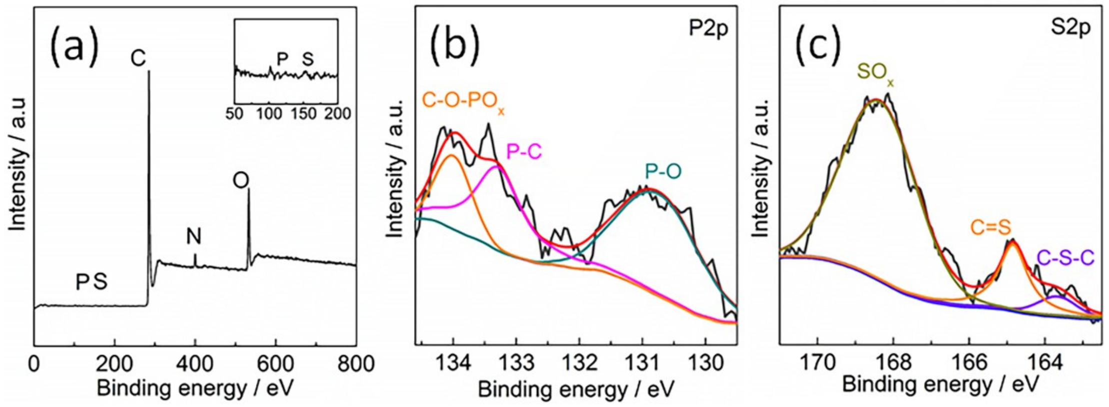

Figure S2. (a) The XPS survey spectra of as-obtained porous carbon sample with high resolution XPS spectra of (b) P 2p and (c) S2p element.

As shown in Figure S2b, there are four characteristic peaks in the P 2p spectra, located at 130.8, 133.2, 133.9and 136.0 eV. And these peaks are ascribed to formation of P-O, P-C, and C-O-PO_x. Figure S2c exhibits the S 2p spectra, associated with three peaks at 163.7 eV (C-S-C), 164.8 eV (C=S) and 168.4 eV (C-SO_x-C), respectively. It verified that the P and S elements were successfully doped into the porous carbon.

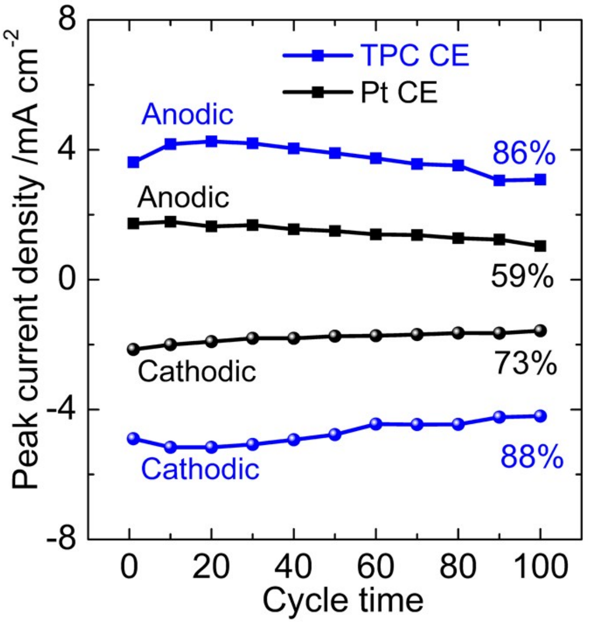

Figure S3. The changes in cathodic and anodic peak current densities of redox couple (I_3^-/I^-) on the TPC and Pt electrode in CV measurement during the 100 scanning cycles.

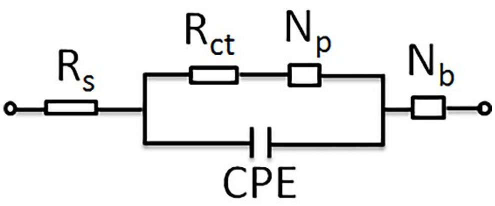

Scheme S1. The equivalent circuit for fitting experimental data in Figure 5b. R_s: Series resistance; R_{ct}: charge transfer resistance; CPE: double-layer capacitance; N_p: Nernst diffusion impedance within the electrode pores; N_b: bulk Nernst diffusion impedance in the electrolyte.

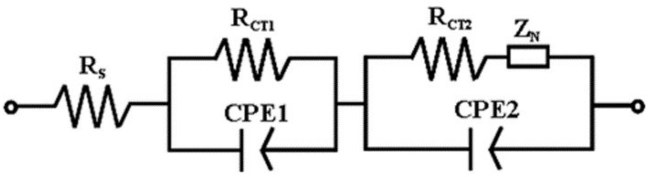

Scheme S2. The equivalent circuit for fitting data in Figure 6b. R_{ct1}: the charge-transfer resistance at the interface of the counter electrode and the liquid electrolyte; R_{ct2}: charge transfer resistance at the TiO_2/dye/electrolyte interfaces; Z_N: Nernst diffusion resistance of the redox couple within the electrolyte; CPE1 and CPE2: double-layer capacitances.
